# A mindfulness-based stress management program and treatment with omega-3 fatty acids to maintain a healthy mental state in hospital nurses (Happy Nurse Project): study protocol for a randomized controlled trial

**DOI:** 10.1186/s13063-015-0554-z

**Published:** 2015-01-31

**Authors:** Norio Watanabe, Toshi A Furukawa, Masaru Horikoshi, Fujika Katsuki, Tomomi Narisawa, Mie Kumachi, Yuki Oe, Issei Shinmei, Hiroko Noguchi, Kei Hamazaki, Yutaka Matsuoka

**Affiliations:** Department of Clinical Epidemiology, Translational Medical Center, National Center of Neurology and Psychiatry, 4-1-1 Ogawa-higashi, Kodaira, Tokyo Tokyo 187-8551 Japan; Departments of Health Promotion and Human Behavior and of Clinical Epidemiology, Kyoto University Graduate School of Medicine/School of Public Health, Yoshida Konoe-cho, Sakyo-ku, Kyoto, 606-8501 Japan; National Center for Cognitive Behavior Therapy and Research, National Center of Neurology and Psychiatry, 4-1-1 Ogawa-higashi, Kodaira, Tokyo 187-8551 Japan; Department of Psychiatric and Mental Health Nursing, Nagoya City University School of Nursing, 1 Kawasumi, Mizuho-ku, Nagoya, 467-0001 Japan; Department of Clinical Research, Translational Medical Center, National Center of Neurology and Psychiatry, 4-1-1 Ogawa-higashi, Kodaira, Tokyo Japan; Correspondence Division, Musashino University, 1-1-20 Shinmachi, Nishitokyo, Tokyo 202-8585 Japan; Department of Public Health, Faculty of Medicine, University of Toyama, 2630 Sugitani, Toyama, 930-0194 Japan

**Keywords:** Anxiety, Behavior therapy, Depression, Fatty acids, Omega-3, Mindfulness, Prevention and control

## Abstract

**Background:**

It is reported that nursing is one of the most vulnerable jobs for developing depression. While they may not be clinically diagnosed as depressed, nurses often suffer from depression and anxiety symptoms, which can lead to a low level of patient care. However, there is no rigorous evidence base for determining an effective prevention strategy for these symptoms in nurses. After reviewing previous literature, we chose a strategy of treatment with omega-3 fatty acids and a mindfulness-based stress management program for this purpose. We aim to explore the effectiveness of these intervention options for junior nurses working in hospital wards in Japan.

**Methods/Design:**

A factorial-design multi-center randomized trial is currently being conducted. A total of 120 nurses without a managerial position, who work for general hospitals and gave informed consent, have been randomly allocated to a stress management program or psychoeducation using a leaflet, and to omega-3 fatty acids or identical placebo pills. The stress management program has been developed according to mindfulness cognitive therapy and consists of four 30-minute individual sessions conducted using a detailed manual. These sessions are conducted by nurses with a managerial position. Participants allocated to the omega-3 fatty acid groups are provided with 1,200 mg/day of eicosapentaenoic acid and 600 mg/day of docosahexaenoic acid for 90 days.

The primary outcome is the change in the total score of the Hospital Anxiety and Depression Scale (HADS), determined by a blinded rater via the telephone at week 26. Secondary outcomes include the change in HADS score at 13 and 52 weeks; presence of a major depressive episode; severity of depression, anxiety, insomnia, burnout, and presenteeism; utility scores and adverse events at 13, 26 and 52 weeks.

**Discussion:**

An effective preventive intervention may not only lead to the maintenance of a healthy mental state in nurses, but also to better quality of care for inpatients. This paper outlines the background and methods of a randomized trial that evaluates the possible additive value of omega-3 fatty acids and a mindfulness-based stress management program for reducing depression in nurses.

**Trial registration:**

Clinicaltrials.gov: NCT02151162 (registered on 27 May 2014).

## Background

Hospital nurses are vulnerable to psychological stress and mental disorders [1]. In early reports, the suicide rate of female nurses was significantly higher than that of average workers, and their life expectancy at the age of 45 years was 26.9 years, which is only one year more than that of miners [2]. In a cross-sectional survey conducted in Cyprus, 65% of nurses reported that their job was stressful, and the prevalence of fatigue was higher in females (93%) than in males (88%) [3].

Consequently, nursing is highly associated with depression. The prevalence of depressive symptoms above a clinical cut-off among hospital-employed nurses is 18% in the United States [4]. Nurses with depression are not only likely to suffer personally, but their illness may also have an impact on the quality of care for patients, through an increase in presenteeism. In terms of the monetary burden of patient care, the costs due to increased falls and medication errors that are caused by presenteeism are estimated at 1,346 USD per nurse annually [5].

A systematic review conducted on psychological distress among hospital nurses in the United States reported that emotional fatigue and burnout in nurses is associated with lack of support from senior management in the form of safety programs, and support from supervisors, respectively [6]. Less-experienced junior nurses need support from senior colleagues and require interventions to empower resilience in order to alleviate their psychological distress.

When considering the possible options for intervention in depression, pharmacological treatments can be discounted because they are not useful as a preventive intervention due to their adverse side effects. Instead, lifestyle interventions, such as exercise and diet, are highly likely to be easily implemented and are promising options. With regard to exercise, according to a systematic review of randomized controlled trials (RCTs), depressive symptoms among patients with a chronic illness and functioning problems were reduced [7]; however, rigorous evidence has not supported exercise as a preventive measure for depression and anxiety symptoms among the elderly [8] or children and young people [9]. In addition, intensive exercise may not be feasible due to the busy schedule of nurses working in hospitals. In contrast, the most feasible interventions may be diet. Omega-3 polyunsaturated fatty acids (PUFAs), in particular eicosapentaenoic acid (EPA) and docosahexaenoic acid (DHA), have been most frequently evaluated for their effects on depression and anxiety disorders. Many previous systematic reviews that have accumulated data from RCTs have been published; however, there are some reviews that show a positive effect of omega-3 PUFAs [10] and others that do not [11]. In addition, these reviews did not focus on preventative interventions.

In a recently published comprehensive meta-analysis [12], omega-3 PUFAs were effective in patients with a diagnosis of major depressive disorder, but not effective at preventing depression in healthy subjects. In this analysis, three parallel design RCTs for the prevention of depression in healthy subjects were assessed, with a follow-up duration of over three months. The studies employed a daily dose of 1.093 g EPA and 0.226 g DHA [13], 3.0 g EPA and 0.6 g DHA [14] or 3.0 g EPA and 0.6 g DHA [15]. However, a high daily EPA dose of between 1,000 and 1,500 mg/d with a ratio of 2:1 DHA is argued as optimal for affective disorders [16]. Based on the current evidence for omega-3 PUFAs as a preventive tool for anxiety and depressive symptoms, we believe that methodologically rigorous trials employing optimal doses of EPA and DHA are still needed.

On the other hand, psychological interventions, such as stress management, problem solving and support should be considered as possible interventions in workplace settings because of their feasibility and accessibility. A Cochrane review [17] has revealed that a psychological intervention to alleviate stress among workers in the workplace can be efficacious according to several small RCTs; however, this review concluded that the methodological quality of these trials was not sufficient, and further methodologically sound trials with a larger sample size are needed to reach firm conclusions.

Among the various types of psychotherapy, mindfulness-based cognitive therapy and meditation have been given particular attention by both clinicians and researchers. In a systematic review, mindfulness meditation programs have shown moderate evidence of improvements in depression, with an effect size of 0.30 (95% confidence interval (CI): 0.00 to 0.59) at post-treatment), when compared with nonspecific active controls [18]; however, the review focused on a clinical sample and therefore was not able to conclude any preventive effects of the psychotherapy. To the best of our knowledge, there has been no systematic review focusing on the prevention of depression and anxiety symptoms in the workplace. Although the efficacy of mindfulness-based cognitive therapy in nurses has been examined in a previous RCT [19], the trial included only a small number of participants and no firm conclusions could be drawn. For this reason, a conclusive RCT is necessary to establish evidence of an effective psychological intervention that can help to maintain a healthy mental state in hospital nurses.

Hence, the present study aims to conduct a methodologically rigorous factorial-design trial with 1:1:1:1 allocation to explore the effectiveness of omega-3 PUFAs, and a mindfulness-based stress management program for maintaining a healthy mental state in hospital nurses.

## Methods/Design

### Trial design

A parallel-arms factorial-design RCT with a 52-week follow-up period has been planned. Participants will be randomly allocated to one of the four intervention arms: 1) mindfulness-based stress management program plus omega-3 PUFAs; 2) mindfulness-based stress management program plus placebo; 3) psychoeducation leaflet plus omega-3 PUFAs and 4) psychoeducation leaflet plus placebo. A total of 30 participants will be allocated to each arm. These interventions will terminate within three months from the registration of the participant. The primary outcome will be the depression and anxiety severity at 26 weeks (rated blindly), assessed using the 14-item Hospital Anxiety and Depression Scale (HADS). After each assessment, an assessor will guess which group the participant has been assigned to, making it possible to examine if the blinding is successful.

### Inclusion criteria

The inclusion criteria for participants are as follows:

1. Aged between 20 and 59 years at entry to the study and of female gender. We will limit participants to the female gender because a previous study investigating the prevention of post-traumatic stress among emergency workers has demonstrated that omega-3 fatty acids are effective in females but not males [20]. This is the first study to examine the effectiveness of omega-3 fatty acids among nurses, therefore we have decided to focus on females to maximize the benefit.
2. Nurses who work in inpatient wards at four general hospitals in the Tama region of Japan, including the National Center of Neurology and Psychiatry Hospital, National Disaster Medical Center, Tokyo Metropolitan Tama Medical Center and Tama-Hokubu Medical Center.
3. Those who are mainly engaged in caring for patients, but not in administrative responsibilities. Thus, head nurses in wards and senior nurses who are mainly engaged in supporting administrative work of the head nurses are excluded. A previous study has shown that senior nurses have less burnout than junior nurses [21], and senior nurses are engaged in administrative work in the hospitals included in our study.
4. Those who have given written informed consent to participate in the study.

### Exclusion criteria

The exclusion criteria are as follows:

1. Plans to take sick leave, leave for other reasons or retirement within 26 weeks from entry to the study;
2. Already engaging in structured psychotherapy (such as cognitive behavioral therapy, interpersonal therapy or brief psychodynamic therapy) at entry;
3. Seeing a physician regularly for the treatment of any mood or anxiety disorders at entry;
4. Taking antidepressants, mood stabilizers, anticonvulsants or antipsychotics at entry;
5. Any history of taking nutrient supplements, including omega-3 fatty acids, for four or more weeks within 52 weeks from entry;
6. Clinical depression, based on a total score of 11 or more on the HADS - Depression Subscale [22] and 15 or more on the Primary Health Care Questionnaire [23];
7. Consumption of fish as the main course of a meal four or more times per a week;
8. Taking anticoagulant drugs at entry or history of stroke or myocardial infarction or
9. Judged ineligible by a clinical research coordinator (CRC) for any reason.

### Procedures

Information about the study will be presented to the head of the hospital, the director of the nursing service department and head nurses in explanatory meetings at the recruiting hospitals. Leaflets with information about the study will be distributed to nurses in hospital wards and interested nurses will be contacted by a CRC. Each nurse will input her data, except the HADS criterion, into an electronic data capturing (EDC) system via the internet, with some assistance by the CRC, and if she satisfies all of the inclusion criteria and none of the exclusion criteria, provisional written informed consent will be obtained. The participants will then be interviewed using the HADS through their mobile phone by a rater located in Kyoto University. If the nurse does not satisfy the HADS exclusion criterion, formal written informed consent will be obtained.

**Figure 1:**
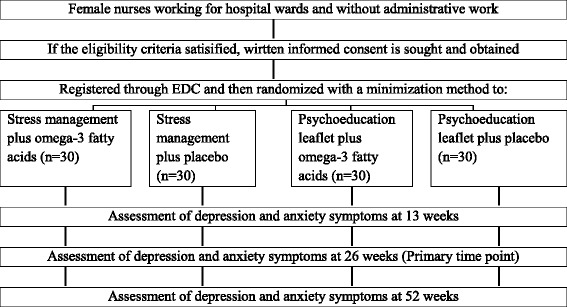
**Flowchart of the trial.** EDC, electronic data capturing.

The participants will be randomly allocated to one of the four intervention arms via the EDC using a minimization method. Minimization is an adaptive stratified allocation system that is used in clinical trials [24]. Minimization can reduce the imbalance between the numbers of patients in each treatment group over possible confounding factors. The participant’s place of work, total score on the HADS of 11 or higher [22], and whether they have been working as a nurse for one year or more will be used as stratification variables (Figure 1).

### Interventions

#### Psychological interventions

In the present factorial-design RCT, the effects of two forms of psychological intervention will be compared: mindfulness-based stress management program and a psychoeducation leaflet.

The stress management program has been developed combining mindfulness-based self-regulation of attention [25] and the cognitive behavioral model of depression adapted from a previously established manual [26]. Although a previous trial of 41 nursing students [27] employed mindfulness meditation with the non-judgmental awareness of moment-to-moment experiences as the central axis [28], we extracted the treatment components of body scan and breathing meditation from mindfulness meditation because these techniques are considered easy to learn and helpful for nurses, in our experience. The stress management program consists of four weekly 30-minute individual sessions and is highly structured, with a detailed manual. The contents of each session are as follows: 1) understanding the cognitive behavioral model of stress and emotion of the participant, 2) reviewing the model and conducting body scanning and breathing exercises, 3) reviewing the results of model and body scanning and then promoting awareness of the cognition of the participant and 4) reviewing the model, providing suggestions about increasing pleasant behaviors and planning a strategy to manage future stress. The feasibility of the program has been examined by employing five nurses as participants in a pilot run, the protocol of which was approved by the institutional review boards of the National Center of Neurology and Psychiatry on 15 November 2013 (approval number: A2013-102). No dropouts due to the program were observed. All the participants reported that the program might be helpful for nurses to manage stress from their work. Some rewordings have been done to the program according to comments from the participants.

The stress management program will be conducted by senior nurses in four individual sessions within the first three months. Senior nurses will have taken a seven-hour workshop, including lectures and role-playing sessions. The detailed manual and videos, including lectures, will be provided to the senior nurses. The senior nurses will be supervised monthly by email once the program commences. All sessions will be audiotaped and 20% of them will be randomly selected and evaluated by two independent researchers to assess the integrity of the program. They will check for adherence to the treatment manual with a checklist for each session.

A leaflet has been prepared for the psychoeducation leaflet groups. This is based on a freely distributed psychoeducation leaflet about healthy mental state written by the Japanese Ministry of Health, Labor and Welfare [29]. The leaflet includes information about checking for signs of stress, relaxation and sleep hygiene, and suggestions for stress management such as exercise, laughter and meeting intimate friends. It also includes a list of consultants for mental health. We have adapted this leaflet to be applicable to our study by adding advice from senior nurses about working without stress. Participants allocated to the leaflet groups will be instructed to read this often, especially when they feel anxious or stressed.

### Supplements interventions

Omega-3 PUFA capsules have been formulated to contain 1,200 mg EPA and 600 mg DHA (Kentech Co. Ltd., Toyama, Japan) according to recommendations from experts [16]. The participants will take the capsules once a day for 90 days from entry into the study. Placebo capsules contain rapeseed oil (47%), soybean oil (25%), olive oil (25%) and fish oil (3%), and have an identical appearance and similar odor (Kentech Co. Ltd., Toyama, Japan).

Adherence to the regimens will be checked among nurses working for the National Center of Neurology and Psychiatry Hospital and National Disaster Medical Center from results of blood tests. At baseline and at the three-month assessment, red blood cells (RBCs) will be obtained from EDTA-anticoagulated blood (Terumo Corporation, Tokyo, Japan), washed twice with saline and frozen at −80°C until analysis. The fatty acid composition of the total phospholipid fraction of the RBCs will be determined as described previously, with slight modifications [30]. Briefly, the total lipid content will be extracted from RBCs according to the method used by Bligh and Dyer [31]. The total phospholipid fraction will be separated by thin-layer chromatography. After transmethylation with HCl-methanol, the fatty acid composition will be analyzed using a gas chromatography system (GC14A Shimadzu Corporation, Kyoto, Japan) equipped with a capillary column DB-225 (length: 30 m; internal diameter: 0.25 mm; film: 0.25 μm; J&W Scientific, Folsom, California, United States); the entire system was controlled using gas chromatography software CLASS-GC10 version 1.3 (Shimadzu Corporation). In addition, to assess adherence to the regimen, all the remaining capsules will be collected from all the participants after the three-month assessment.

Participants who decide to leave the allocated intervention due to adverse events or any other reason within the first 13 weeks will be classified as dropouts from the intervention, but will still be asked to complete the assessments. Participants allocated to the leaflet and placebo capsule groups will not be allowed to take the stress management program and omega-3 PUFA capsules respectively, after 26 weeks. However, they will be allowed to take these interventions thereafter, and information will be collected from these participants.

### Assessment measures

Information about depression and anxiety symptoms (primary outcome), insomnia, burnout, presenteeism, quality of life, sick leave, consultation about the mental state of the participant and oxidative stress will be collected at baseline, three months, six months (primary time point) and 12 months from registration for each participant (Table 1).

**Table 1:** **Schedule for the assessments**

		**Entry**	**7 to 21 days after entry**	**13 weeks from entry (allowing for subsequent 14 days)**	**26 weeks from entry (allowing for subsequent 30 days)**	**52 weeks from entry (allowing for subsequent 30 days)**
Kyoto University	HADS assessed through telephone	x		x	x	x
	Allocation guessed by raters			x	x	x
CRC	Informed consent, registration to EDC, information about allocated group	x				
	Socio-demographic variables (such as age, academic status, marital status and partners)	x				
	Concentrations of omega-3 fatty acids in membrane of RBC, oxidative stress, and antioxidant status	x		x		
		(2 hospitals)		(2 hospitals)		
Participants’ input through EDC	PHQ-9	x		x	x	x
	GAD-7	x		x	x	x
	ISI	x		x	x	x
	MBI	x		x	x	x
	BSI	x	x	x	x	x
	HPQ	x		x	x	x
	EQ-5D	x		x	x	x
	Numbers of incidents and accidents	x		x	x	x
	Numbers of visits to clinics	x		x	x	x
	Numbers of absent days due to any reason	x		x	x	x
	History of taking psychotropic drugs	x		x	x	x
	Adverse events		x	x	x	x

BSI, Bradford Somatic Inventory; CRC, clinical research coordinator; EDC, electronic data capturing; EQ-5D, EuroQol; GAD-7, Generalized Anxiety Disorder 7-item scale; HADS, Hospital Anxiety and Depression Scale; HPQ, World Health Organization Heath and Work Performance Questionnaire; ISI, Insomnia Severity Index; MBI, Maslach’s Burnout Inventory; PHQ-9, Patient Health Questionnaire; PRIME-MD, Primary Care Evaluation of Mental Disorders.

### Primary outcome measure

The primary outcome will be the total depression and anxiety severity changes at week 26 (rated blindly), assessed using the 14-item HADS [32,33] through their mobile phone by a blinded rater located at Kyoto University. All the patients will be requested not to reveal the allocated treatment to the assessors, in order to keep the assessors’ blindness to the groups.

The total score of the HADS (HADS-T) ranges from 0 to 42, with higher scores indicating more symptoms. The HADS has two sub-scores, each ranging from 0 to 21: HADS-D (depression) and HADS-A (anxiety). The defined cutoffs were eight or higher for mild to moderate symptoms and 11 or higher for severe symptoms of anxiety or depression. The reliability and validity of the Japanese version of the HADS has been confirmed, with Cronbach’s alpha values of the scale at 0.79 for the HADS-D and at 0.77 for the HADS-A [22]. The total score will be used as a measure of the severity of depression and anxiety symptoms in the present study.

#### *Secondary outc*o*me measures*

All other self-reported measures will be collected through the EDC system, where the participants can enter their own data at home. Participants will be notified at 13, 26 and 52 weeks to fill in the assessment questionnaires within the following 14, 30 and 30 days through the EDC system, respectively.

### Depression and anxiety symptoms

The HADS will be administered at baseline, 13 and 52 weeks as secondary outcomes, as well as at 26 weeks (the primary outcome).

### Major depressive episode

A current major depressive episode, according to (the Diagnostic and Statistical Manual of Mental Disorders, 4th. Edition (DSM-IV) criteria, will be ascertained using the Primary Care Evaluation of Mental Disorders (PRIME-MD) algorithm in the depression module of the Patient Health Questionnaire (PHQ-9) [23] at baseline, 13, 26 and 52 weeks. The PHQ-9 was developed as a self-report version of the PRIME-MD that aims at a DSM-IV diagnosis of several common mental disorders. The PHQ-9 has been used as an assessment of major depressive disorder, according to DSM-5 [34]. The PHQ-9 consists of the nine diagnostic criteria items, which make a total score range between 0 and 27. Good test-retest reliability (intraclass correlation coefficient (ICC) = 0.92) [35] and internal consistency reliability (Cronbach’s alpha = 0.89) [23] have been reported. The Japanese version has been established through back-translation [36].

### Anxiety

The Generalized Anxiety Disorder 7-item scale (GAD-7) [37] will be used to assess the severity of anxiety symptoms in the participants at baseline, 13, 26 and 52 weeks. The GAD-7 was developed to screen for and evaluate the severity of generalized anxiety disorder (GAD) in the general population. The GAD-7 consists of seven items and can give a diagnosis according to DSM-IV and DSM-5. The internal consistency and test-retest reliability of the GAD-7 were a Cronbach’s alpha of 0.92 and intraclass correlation of 0.83, respectively [37]. Total scores between five and nine, 10 and 14, or 15 and 21 indicate mild, moderate or severe anxiety symptoms, respectively. According to a recently published systematic review, the GAD-7 was the best performing test for GAD, with a positive likelihood ratio of 5.1 (95% CI: 4.3 to 6.0) and a negative likelihood ratio of 0.13 (95% CI: 0.07 to 0.25) [38]. A Japanese version of the GAD-7 has been developed [39] and the agreement rate of diagnosis compared to the Mini International Neuropsychiatric Interview was reported as satisfactory.

### Burnout

The Maslach’s Burnout Inventory (MBI) [40] will be used to assess burnout in nurses. The MBI is a tool for detecting and measuring the severity of burnout syndrome. It is a 22-item questionnaire that assesses the degree of burnout in terms of three subscales: emotional exhaustion (EE), depersonalization (DP) and personal accomplishment (PA). EE represents feelings of being emotionally exhausted and having a lack of energy, DP represents feelings of impersonal responses towards recipients of the service and PA represents feeling of incompetence. The original English version has a good validity and reliability, with a Cronbach’s alpha of 0.71 to 0.90 [41,42]. The Japanese version also has good reliability, with a Cronbach’s alpha of 0.77, 0.76 and 0.60 for EE, DP and PA, respectively [43].

### Insomnia

The Insomnia Severity Index (ISI) [44,45] will be used to assess insomnia at baseline, 13, 26 and 52 weeks. The ISI is now considered a standard global measure for assessing the severity of insomnia and is used in many studies [46,47]. Total scores between eight and 14 and 15 and 28 indicate subthreshold insomnia and clinical insomnia, respectively. The internal consistency of the ISI has a Cronbach’s alpha of 0.90 in a community population [48]. In a clinical population, a difference in score of −8.4 points (95% CI: −7.1 to −9.4) was associated with a moderate improvement, as rated by an independent assessor after treatment [48]. The Japanese version has been validated [49].

### Somatic symptoms

The Bradford Somatic Inventory (BSI) will be used to measure somatic symptoms at baseline, 13, 26 and 52 weeks. The BSI is a 44-item questionnaire for female subjects about symptoms experienced in the previous month, which was designed to detect physical symptoms commonly related to depressed patients [50]. We selected the BSI as a measurement for somatic symptoms because there are no other assessment tools for this purpose in the Japanese language. The original English version of the BSI was translated into Japanese by two Japanese psychiatrists (NW and YM) and back-translated into English by one psychiatrist (TAF). The translations were then checked by the original author of the BSI (Professor David B Mumford). The number of positive items in the BSI will be used as a measure of the severity of somatic symptoms in this study. Data from the BSI will be collected through the EDC on days seven to 21 from entry into the study to check the test-retest reliability.

### Presenteeism

The World Health Organization Heath and Work Performance Questionnaire (HPQ) will be used to assess presenteeism at baseline, 13, 26 and 52 weeks. The HPQ is a self-report instrument designed to estimate the workplace costs of health problems in terms of self-reported reduced job performance (presenteeism). The HPQ measures of presenteeism can be used to calculate two scores: the absolute presenteeism score, obtained by multiplying the respondent’s response to the second question by 10, and the relative presenteeism score, obtained by dividing the first response by the second response, then multiplying by 100. Absolute and relative presenteeism scores will be used in this study.

The validity of the scale has been confirmed in previous studies [51,52]. The Japanese version of the HPQ was used in the World Mental Health Survey in Japan [53]. The area under the curve values for absolute and relative presenteeism in relation to future absences due to mental disease were 0.71 (95% CI: 0.62 to 0.80) and 0.65 (95% CI: 0.55 to 0.75), respectively [54].

### Quality of life

The EuroQol (EQ-5D) [55] will be used to assess health-related quality of life (QoL) at baseline, 13, 26 and 52 weeks. The EQ-5D is a standardized instrument used as a measure of health outcome and is applicable to a wide range of health conditions and treatments. The five domains include mobility, self-care, usual activities, pain and/or discomfort, and anxiety and/or depression. There are three levels of severity: no problems, some or moderate problems and severe or extreme problems. Each pattern of responses is allocated to an individual utility score, which ranges from 0 (death) to one (perfect health). The Japanese version has been developed [56]. The test-retest reliability of the Japanese version was 0.996, and Cronbach’s alpha was 0.827 [57].

### Oxidative stress

Oxidative stress and antioxidant levels will be evaluated at the baseline and three-month assessments among the nurses working for the National Center of Neurology and Psychiatry Hospital and National Disaster Medical Center. Oxidative stress is the imbalance between oxidative stress and antioxidant defenses. High oxidative stress or low antioxidant status are becoming increasingly recognized as biological mechanisms associated with poor health outcomes and the progression of a wide range of diseases [58,59]. An association between depression and oxidative stress has been reported in a recently published meta-analysis [60]; however, the conclusions seem inconsistent due to large heterogeneity among the included studies, with an I^2^ of 80% and possible selection bias as the studies did not cover a broad range of depression. Moreover, the meta-analysis included only cross-sectional studies; therefore, associations between changes in depression and oxidative stress remain unclear.

In the present study, we will use reactive oxygen metabolite-derived compounds (d-ROMs) and biological antioxidant potential (BAP) tests [61] to evaluate oxidative stress and antioxidant levels, respectively, as used in previous studies [62,63].

### Adverse events

An adverse event is defined as any unwanted or unintended signs (including laboratory exams, suicidal and self-harm behaviors and very unstable mental states), symptoms or disease seen in trial participants, regardless of the causal relationship with the study intervention. Information about any possible adverse events will be collected during the intervention period through the EDC. Participants will be encouraged to input these data via emails at fixed intervals. Information about the severity and degree of adverse events affecting normal life will also be collected. When the severity of severe for adverse events or the degree affecting usual life of moderate or severe due to adverse eventsis presented, the participant will be contacted through telephone or email by a CRC, and termination of the intervention will be discussed.

Adverse events will be classified into major adverse events if they may lead to death or to enduring severe impairment, depending on participants’ conditions and circumstances. Major adverse events shall be reported to the relevant section of the Ministry of Health, Labor and Welfare, as well as to the heads of all research centers and recruiting hospitals.

### Other outcomes

Information about sick leave, consultations on personal mental state and about discontinuation of the allocated interventions will be collected through the EDC.

### Sample size

The sample size was based on a power analysis conducted for the HADS scores. There are no previous reports associated with the stress management program, in terms of nurse-to-nurse interventions, that can contribute to the power calculation of our trial. Based on trials that have used nurses as therapists and the HADS as the primary outcome, one trial has examined the efficacy of cognitive behavioral therapy conducted by nurses for depression in the elderly after lumber fracture operations, and a difference of 3.7 was found compared with treatment as usual [64]. To the best of our knowledge, there have been no reports assessing treatment with omega-3 fatty acids for depression or anxiety in nurses. One systematic review and meta-analysis has shown that treatment with omega-3, ≥60% EPA and ≤40% DHA led to an effect size of −0.026 (95% CI: −0.200 to 0.148), when compared with a placebo [65]. Therefore, the mean difference in the HADS scores was estimated to be 4 ± 6 (SD) between the stress management program and leaflet groups, and between the omega-3 fatty acids and placebo groups. To detect a significant difference at *P* = 0.05 (two-tailed) with a power of 0.9, it was calculated that 48 patients would be required for each of the arms in both comparisons. Thus, allowing for a 20% dropout rate, 60 participants would need to be recruited per group. In the present factorial-design RCT, 30 participants would be necessary for each of the four groups.

### Data management and analysis

All of the participants who are randomized at baseline will be included in the primary analyses (intention-to-treat (ITT)). First, a descriptive analysis of the variables at baseline will be performed. We do not plan any statistical tests to detect a difference at baseline among the trial arms because we aim to avoid multiple tests, and the decision to adjust for baseline data in RCTs should not be determined by whether baseline differences are statistically significant [66]. However, when clinically important differences at baseline are noted from a clinician’s point of view, a sensitivity analysis will be performed by adjusting for all such possible confounds.

Second, for the primary outcome and all the continuous outcomes assessed at 26 and 52 weeks, differences among groups will be examined using a mixed-model repeated measures analysis. When missing data exist for categorical variables, which are all negative outcomes, an ITT principal will be applied by assuming that all dropouts did not satisfy the outcomes. However, per protocol analyses, where only data from completers are incorporated, will also be presented according to a recent recommendation [67]. We also plan to calculate a number needed to treat (NNT) with a 95% CI if a statistically significant difference among the groups is observed in dichotomous data.

A *P* value of <0.05 will be set to test the null hypothesis for all analyses. Although we understand that this decision might lead to errors in multiple tests, the present trial is the first of its kind to examine prevention interventions in this type of workplace; therefore, we have considered that avoiding beta errors is preferable over alpha errors. However, 95% CIs will always be presented for differences among groups. We will not perform interim analyses to examine the study hypotheses.

### Sensitivity analysis

Per protocol analyses, where only data from completers are incorporated to an analysis of covariance that adjusts for baseline data, will be performed as sensitivity analyses for all the continuous outcomes.

### Data monitoring

The trial will be supervised by the Data Monitoring and Safety Management Committee. The committee consists of three independent experts in research on psychotherapy and nutrient interventions and research methodology. Data regarding all outcomes will be sent to the committee twice a year and inspected. The members of the committee are independent from the sponsor and competing interests of the present study.

### Publication policy

The protocol and the results of all outcomes will be published in a peer-reviewed medical journal. The results shall be reflected in treatment guidelines and systematic reviews.

### Study period

The study period of this trial will be between June 2014 and March 2016, with the participant entry period being between June 2014 and March 2015.

### Ethical issues

The present study complies with the ethical guidelines for clinical studies published by the Japanese Ministry of Health Labor and Welfare, as well as the ethical principles established for research on humans stipulated in the Declaration of Helsinki and further amendments thereto.

The study protocol has been approved by the institutional review boards of the National Center of Neurology and Psychiatry on 16 May 16 2014 (approval number: A2014-017) and of Toyama University on 16 June 2014 (approval number: 26–24). If important protocol modifications such as changes to eligibility criteria, outcomes or analyses are needed for any reason, the investigators will communicate this with the review boards.

Written informed consent will be obtained from all participants included in this study. Participants will be informed that they can withdraw from the study at any time without stating the reason, and that their withdrawal will never lead to refusal of any other services. Data for each participant will be handled with sequentially allocated numbers to keep participant’s confidentiality. Participants’ personal information will also be anonymized with sequentially allocated numbers.

## Discussion

To the best of our knowledge, this study represents the first study to investigate the effectiveness of preventive interventions on depression and anxiety symptoms among hospital nurses. In addition to these symptoms, approximately one in four nurses who are shift workers in Japan suffer from shift work disorder [68], which is characterized by excessive sleepiness and/or insomnia associated with their shift work schedule [69]. The high prevalence of these common psychiatric symptoms is probably due to the high responsibility, need for high levels of expertise and relatively low reward associated with the nursing profession. A significant association was found between depressive symptoms and effort-reward imbalance, with an odds ratio (OR) of 10.8, and between esteem-reward imbalance, with an OR of 3.2 in nurse managers working in hospitals in France [70].

It would be hard to restructure the proportion of hospital workers and increase the number of nurses, or to reduce the responsibility and increase the salary of nurses. However, realistic interventions are needed to empower resilience in order to alleviate psychological distress among nurses. Lifestyle interventions, such as diet and psychotherapeutic approaches, are highly desirable options. When an effective preventive intervention has been established, the results may not only lead to the maintenance of a healthy mental state in nurses, but also a better quality of care for inpatients. We believe that the present study will be able to contribute to this goal.

The present study is not without methodological limitations. First, a previous observational study with a large sample size has reported that an increase in a nurses’ workload by one patient, and no bachelor’s degree-level education led to an increase in preventable hospital deaths [71]. We do not plan to collect information about patient outcomes, because inpatients from the hospitals included in the study are usually discharged within approximately 14 days, and many nurses are involved in the care of each patient. Therefore, we have decided *a priori* not to investigate the efficacy of interventions on patient outcomes.

Second, some essential organizational characteristics, including decision-making latitude and quality and quantity of support from peer nurses, are not considered in the study. All of the hospitals that are enrolled in the study are funded by national or local government and located in the capital city of Japan. Neither private nor rural hospitals are included. These may have an effect on the mental health of nurses and therefore these impose a negative impact on the applicability of findings from the study. However, the methodology of quantitatively measuring decision-making latitude, and quality and quantity of support from peer nurses in various disciplines has not been confirmed [6]. The hospitals in this study are core general hospitals in the district and we believe that they are representative among hospitals in developed countries.

Third, because fish consumption is higher in Japan than in other countries, one may doubt the applicability of the findings about omega-3 PUFAs from the present study. A high consumption of fish has been reported to be correlated with a lower countrywide prevalence of major depression, according to a study published in 1998 [72]. However, the Japanese diet has been changing to a more Western diet similar to countries in other parts of the world. Moreover, we have excluded participants who consume fish as the main course of a meal four or more times per week. Therefore, we believe that our findings can be applied to the other countries.

In conclusion, this paper outlines the background and methods of a randomized trial evaluating the promising interventions for maintaining a healthy mental state in hospital nurses.

### Trial status

The randomized trial is currently in the phase of participant enrolment and follow-up.

## Abbreviations

BAP: Biological antioxidant potential
BSI: Bradford Somatic Inventory
CI: confidence interval
CRC: Clinical research coordinator
DHA: Docosahexaenoic acid
DSM: Diagnostic and Statistical Manual of Mental Disorders
EPA: Eicosapentaenoic acid
EDC: Electronic data capturing
EQ-5D: EuroQol
GAD-7: Generalized Anxiety Disorder 7-item scale
HADS: Hospital Anxiety and Depression Scale
ICC: Intraclass correlation coefficient
ISI: Insomnia Severity Index
ITT: Intention-to-treat
MBI: Maslach’s Burnout Inventory
NNT: Number needed to treat
OR: Odds ratio
PHQ-9: Patient Health Questionnaire
PUFA: Polyunsaturated fatty acid
PRIME-MD: Primary Care Evaluation of Mental Disorders
RCT: Randomized controlled trial
d-ROM: Reactive oxygen metabolites-derived compound
RBCs: Red blood cell
HPQ: World Health Organization Heath and Work Performance Questionnaire
